# Burn Injuries in Patients with Epilepsy: A Retrospective Case Series with Focus on Risk Factors: Epilepsy-related Burn Injuries

**DOI:** 10.31661/gmj.vi.3880

**Published:** 2025-06-29

**Authors:** Xiong Ziting, Li Dan, Zhou Jianwen, Huang Zhenjia

**Affiliations:** ^1^ Department of Dermatology, Chengdu Seventh People’s Hospital, Chengdu, China; ^2^ Department of Burn and Plastic Surgery, Chengdu Second People’s Hospital, Chengdu, China

**Keywords:** Epilepsy, Burn Injury, Seizure Frequency, Antiseizure Medications, Injury Severity

## Abstract

**Background:**

Burn injuries in patients with epilepsy represent a significant public health issue. This study aimed to investigate the demographic and clinical characteristics, causes, and severity of burn injuries in patients with epilepsy and to explore associations with epilepsy-related factors.

**Materials and Methods:**

We conducted a retrospective analysis of 42 patients with epilepsy who sustained burn injuries and were admitted to our hospital between 2015 and 2023. Data on patient demographics, burn type and severity, total body surface area (TBSA), seizure type and frequency, and antiseizure medication (ASM) adherence were collected and analyzed.

**Results:**

Among the 42 patients (28 males and 14 females; mean age 35.6 ± 12.1 years), the most common causes of burns were direct flame (38.1%) and hot liquids (28.6%). Most burns occurred at home (78.6%) during daily activities. Generalized tonic-clonic seizures were the most frequent type (59.5%). Patients with frequent seizures (1/month) and those with poor ASM adherence experienced more severe burns and longer hospital stays (P0.01).

**Conclusion:**

Patients with epilepsy are at increased risk of burn injuries, especially in the context of uncontrolled seizures. Effective seizure management may play a key role in reducing the severity of such injuries and associated complications.

## Introduction

Burn injuries are among the most severe types of traumas due to their lasting physical and psychological effects. Individuals with epilepsy are at a significantly elevated risk of sustaining such injuries, particularly when seizures occur in environments that are not seizure-safe. Although the exact prevalence of burn injuries in this population is not well established, current evidence suggests a higher incidence compared to the general population [1]. The etiologies of burn injuries in epileptic patients vary and include exposure to open flames, scalds from hot liquids, electrical sources, and chemicals. These injuries frequently occur during routine activities—such as cooking or bathing—when a sudden seizure can result in accidental contact with hazardous elements [2][3]. Several factors influence both the likelihood and severity of burn injuries in this group. These include the type and frequency of seizures, adherence to antiseizure medication (ASM), and the presence of supportive environments. Identifying and addressing these risk factors is essential for implementing effective preventive strategies [4].

Despite increasing awareness of the risk of burn injuries among people with epilepsy, limited studies have comprehensively examined targeted prevention approaches. This study aims to address that gap by analyzing the characteristics of burn injuries, identifying associated risk factors, and evaluating the role of preventive measures in a cohort of patients with epilepsy.

### Objectives

1. To analyze the demographic characteristics of patients with epilepsy and burn injuries.

2. To identify the most common causes and circumstances of burn injuries in these patients.

3. To evaluate the association between epilepsy characteristics (seizure type and frequency) and burn severity.

## Materials and Methods

This retrospective study included 42 patients diagnosed with epilepsy who sustained burn injuries and were admitted to the Burn Unit of our hospital between January 2015 and December 2023. Inclusion criteria were a confirmed diagnosis of epilepsy by a neurologist and documentation of burn injury related to a seizure event. Data were collected from patients' medical records, including age, gender, cause and severity of burn, total body surface area (TBSA) burned, site and time of injury, seizure type and frequency, adherence to antiseizure medications (ASMs), and comorbidities.

Burn severity was classified according to the depth and extent of burns, and clinical outcomes were measured by length of hospital stay, surgical interventions, and complications.

Statistical analysis was performed using SPSS software (version 25.0, IBM Corp, Armonk, NY, USA). Descriptive statistics summarized patient characteristics. Chi-square and t-tests were used to assess associations between variables, with p<0.05 considered statistically significant.

## Results

**Table:** Table1. Demographic and Clinical Characteristics of the 42 Patients with Epilepsy and Burn Injuries

Variable	Value
Number of patients	42
Age (mean ± SD)	35.6 ± 12.1 years
Male sex	28 (66.7%)
Burn location	Home (78.6%)
Main activity during injury	Daily routines
Cause of burn	Flame (38.1%), Hot liquid (28.6%), Electrical (19.0%)
Type of epilepsy	Generalized tonic-clonic (59.5%), Focal (23.8%)
Seizure frequency >1/month	27 (64.3%)
Mean TBSA	Variable; from <10% to >30%
ASM adherence	Associated with lower severity and shorter stays

A total of 42 patients were included in the analysis, with a mean age of 35.6 ± 12.1 years. The majority were male (66.7%). Most injuries occurred at home (78.6%), primarily during routine activities. The leading causes of burn injuries were direct flame (38.1%), hot liquids (28.6%), and electrical sources (19.0%).

Regarding epilepsy characteristics, 25 patients (59.5%) had generalized tonic-clonic seizures, while 10 patients (23.8%) experienced focal seizures. Seizure frequency varied, with 64.3% experiencing more than one seizure per month. Burn severity ranged from mild (TBSA<10%) to severe (TBSA>30%). Patients with frequent seizures (>1/month) had significantly more severe burns (P<0.01). Those who adhered to regular ASM therapy experienced less severe burns and had shorter hospital stays. The detailed demographic, clinical, and burn-related characteristics of the patients are presented in Table-1.

## Discussion

Burn injuries caused by epileptic seizures represent a significant health concern, particularly in developing countries, where epilepsy is often underdiagnosed and undertreated. Our findings highlight the clinical characteristics, causes, and outcomes of burn injuries in patients with epilepsy over an eight-year period. This study showed that 42 patients with epilepsy sustained burn injuries, with a higher prevalence among men and an average age in the fourth decade of life. These demographic characteristics are in line with previous reports indicating that epilepsy-related burns are more common in young and middle-aged adults, potentially due to greater engagement in daily household or occupational activities [5][6][7][8].

The upper limbs were the most frequently involved area (42.9%), followed by the lower limbs and trunk. These findings are consistent with previous studies, where limbs were more likely to be injured due to falling or contact with hot objects during seizures [9][10]. In our study, scald injuries and flame burns were the most common causes of injury. Similar patterns have been reported in the literature [11][12][13], suggesting that common domestic hazards such as cooking with open flames or hot liquids pose substantial risks to patients prone to seizure episodes. Notably, a significant proportion of our patients (59.5%) sustained burns while cooking.

Importantly, the majority of patients (85.7%) in our study had a history of uncontrolled seizures.

This is a consistent observation with many other studies [7][9][14], indicating that poor seizure control remains a major risk factor for burn injuries. Factors such as nonadherence to antiepileptic medications, lack of regular follow-up, and poor awareness of seizure triggers may contribute to this situation.

Moreover, 26.2% of the patients had a history of previous burn injuries, highlighting the recurrent nature of trauma in this population.

The average TBSA in our patients was 16.5%, and 52.4% required surgical intervention. Although the majority of patients were managed conservatively, the high rate of surgical needs and the presence of one mortality (2.4%) reflect the potentially serious consequences of such injuries.

These outcomes align with previous reports that emphasize the high morbidity associated with epilepsy-related burns, especially when immediate care is delayed [9][12][15].

Comparative data from studies conducted in other countries also support our findings. For instance, similar demographic and clinical patterns were reported in a retrospective study from India, where epilepsy-related burns were often due to kitchen accidents and affected mainly the upper limbs [16].

In contrast, some European studies reported lower rates of flame burns and a higher prevalence of injury in institutionalized patients, likely reflecting differences in living environments and supervision levels [17].

These variations suggest that cultural practices, domestic settings, and public health infrastructure may influence the type and severity of injuries.

From a socioeconomic perspective, epilepsy-associated burns can lead to significant physical and psychological disabilities, loss of income, and high healthcare expenditures, especially in cases requiring multiple surgeries or rehabilitation. This burden can be exacerbated in low-resource settings where specialized burn care is limited.

Despite the valuable insights provided by this study, certain limitations must be acknowledged.

As a single-center retrospective study, the findings may not be generalizable to broader populations. Additionally, detailed data on seizure types, medication adherence, and psychosocial conditions were not available. Future prospective studies with larger cohorts and qualitative assessments of patient behavior and safety practices could provide more comprehensive understanding.

## Conclusion

Patients with epilepsy are at an increased risk of sustaining burn injuries, particularly during episodes of uncontrolled seizures. These injuries often result in significant morbidity, including the need for surgical intervention and prolonged hospitalization. Our findings highlight the importance of seizure control in reducing the severity of burn outcomes. Collaborative care between neurology and burn units may contribute to improved management of these high-risk patients and should be considered in clinical practice.

## Conflict of Interest

The authors declare that they have no conflict of interest.
